# Age-Related Cognitive Decline in Substance Use Disorder: Impact of Prolonged Substance Consumption

**DOI:** 10.1192/j.eurpsy.2025.638

**Published:** 2025-08-26

**Authors:** C. Roncero, D. Remón-Gallo, L. Aguilar, A. Alvarez-Navares, M. Peña, A. Fernandez-Parra, J. Perez, A. Gonzalez-Sanchez

**Affiliations:** 1 Health Science, European University Miguel de Cervantes, Valladolid; 2 Psychiatry, University of Salamanca; 3 Neurosicence, Institute of biomedicine (IBSAL), Salamanca, Spain

## Abstract

**Introduction:**

The consumption of alcohol, cannabis, cocaine, or heroin causes alterations in the central nervous system, affecting mood, perception, and behaviour. Despite the harmful effects of these substances, they remain widely used. Younger individuals tend to consume cannabis and cocaine, while older adults more commonly use alcohol and prescription medications. Ageing brings changes in consumption patterns and has both physical and mental health consequences

**Objectives:**

This study aims to analyze how age influences the clinical characteristics of patients with Substance Use Disorder (SUD), comparing differences between older and younger users.

**Methods:**

A total of 297 SUD patients participated in this study. They were divided into two groups: those aged 55 and older (G1) n= 88, and those younger than 55 (G2) n= 209. The SF-36 questionnaire was used to assess quality of life, the BIS-11 for impulsivity, the ASRS v1.1 for ADHD, the STAI-R for anxiety, and the AQ for autistic traits. All participants provided informed consent, and the study adhered to ethical guidelines.

**Results:**

G1 showed better social functioning (SF-36) but a significant physical decline compared to G2. G1 also demonstrated lower levels of impulsivity (BIS-11), aggression, anxiety (STAI-R), and ADHD symptoms (ASRS), though higher autistic traits (AQ) were observed in G1.

**Conclusions:**

Ageing reduces impulsivity, aggression, anxiety, and ADHD symptoms in individuals with SUD, but worsens physical health and may increase social isolation and autistic traits. These findings underscore the need to adapt SUD treatments according to age, addressing both physical and psychosocial challenges specific to each group.

**Disclosure of Interest:**

None Declared

